# A Comprehensive Evaluation of Health-Related Life Quality Assessment Through Head and Neck, Prostate, Breast, Lung, and Skin Cancer in Adults

**DOI:** 10.3389/fpubh.2022.789456

**Published:** 2022-04-15

**Authors:** Shirin Jalili, Ramin Ghasemi Shayan

**Affiliations:** ^1^Department of Surgical Technology, Islamic Azad University of Sarab, Sarab, Iran; ^2^Department of Radiology, Paramedical Faculty, Tabriz University of Medical Sciences, Tabriz, Iran

**Keywords:** cancer, health care, assessment tools, life quality approach, life quality and expectancy

## Abstract

Health assessment data assists the well-being and patient care teams' process in drawing up a care and assistance plan and comprehending the requirements of the patient. Comprehensive and precise data about the Quality of Life of cancer patients play a significant part in the development and organization of cancer patient care. Quality of Life has been used to mean a variety of various things, such as health situation, physical function, symptoms, psychosocial modification, well-being, enjoyment of life, and happiness. Chronic diseases such as cancer are among the disorders that severely affect people's health and consequently their Quality of Life. Cancer patients experience a range of symptoms, including pain and various physical and mental conditions that negatively affect their Quality of Life. In this article, we examined cancer and the impact that this disease can have on the Quality of Life of cancer patients. The cancers examined in this article include head and neck, prostate, breast, lung, and skin cancers. We also discussed health assessment and the importance and purpose of studying patients' Quality of Life, especially cancer patients. The various signs and symptoms of the disease that affect the Quality of Life of patients were also reviewed.

## Introduction

People's health is a goal of common, financial, conservational aspects, and personal aspects. Besides additional issues, health knowledge is one of the most significant causes of well-being (1). A health assessment is usually comprised of queries, to which patients reply, that inquiries around individual accounts, dangers, life-changing actions, well-being aims, and general well-being. Health assessment data helps well-being and patient care teams' process, provide a plan of care and assistance, and comprehend the requirements of patients (2). Collecting data to make valuable and essential changes to people's health is the chief directive of assessing well-being care requirements (3). Reviewing and researching patients' quality of life can give us essential information about patients' responses to cancer and cancertreatments, and in the communication of diverse responses and the general QoL, the data gained can also affect the appropriate care options (4).

The idiom Quality of Life is extensively used to evaluate quality of life and well-being matters (5). The purpose of studying quality of life is to gain the essential data for political and health plans (6). Emotional suffering in cancer patients is more profound than physical suffering (7–9). Quality of Life has various more profound meanings such as health situation, physical functions, psychosocial alteration, well-being, life pleasure, and happiness. This idiom was intended to narrow the emphasis to the effects of well-being, disease, and cure on quality of life (10). The emotional performance of cancer patients has been widely studied due to its excessive influence on patients' quality of life. Nervousness and depression are disturbing and limiting signs in patients with cancer (11). Cognitive function is another area that is affected by cancers. Cognitive dysfunction includes reduced capacity, particularly in memory, that harms the function and focus of the patient (12–14). The importance of diagnosing and treating depression has been known not only to imrpove quality of life, but since it might negatively affect compliance with cure, the length of time in the hospital and capacity for self-care (11, 15). Cancer and its treatment cause physical incapacities and psychological and social injury that can be diagnosed and identified in order to improve the quality of life relating to well-being (16, 17). Health-related quality of life (HRQoL) raises multidimensional valuation that contains at least the physical, emotional, and social areas and might contain further areas such as cognitive functioning, sexuality, and spirituality. Some instances are role functioning, social functioning, sense of health, pain, and fatigue (18). According to research, the side effects of treatment depend on the person's condition and type of cancer and its treatment affects the patient's QoL (19–21). There are variances between HRQoL and QoL–QoL is a comprehensive idea covering all aspects of social life; however, HRQoL emphasizes the effects of disease and the influence of that disease's treatment on QoL. HRQoL is occasionally confused with health position or functional position (22). Imaging systems and their uses can evaluate all the identifiable characteristics of the cancers mentioned in this article (23). In this article, we discuss the quality of life of patients with head and neck, prostate, breast, lung, and skin cancers that have been affected by their disease, mainly concentrating on numerous factors associated with QoL. These analyses were done by overall and comprehensive evaluation of contemporary studies plus valuable data discussions and vital relativity. In this article, due to limited resources, we examined some main scales through the quality of life, such as physical functioning, emotional functioning, social functioning, cognitive functioning role functioning, nausea and vomiting, appetite loss, fatigue, pain, dyspnea, and diarrhea in the adult age group. It is worth mentioning that due to the importance of these five cancers (Head and Neck, Prostate, Lung, and Skin), their prevalence among adults according to diverse statistics, and lack of sufficient data in these areas, they were analyzing ed at priority which undoubtedly will play an important role accordingly.

## Head and Neck Cancer

**Figure 1:**
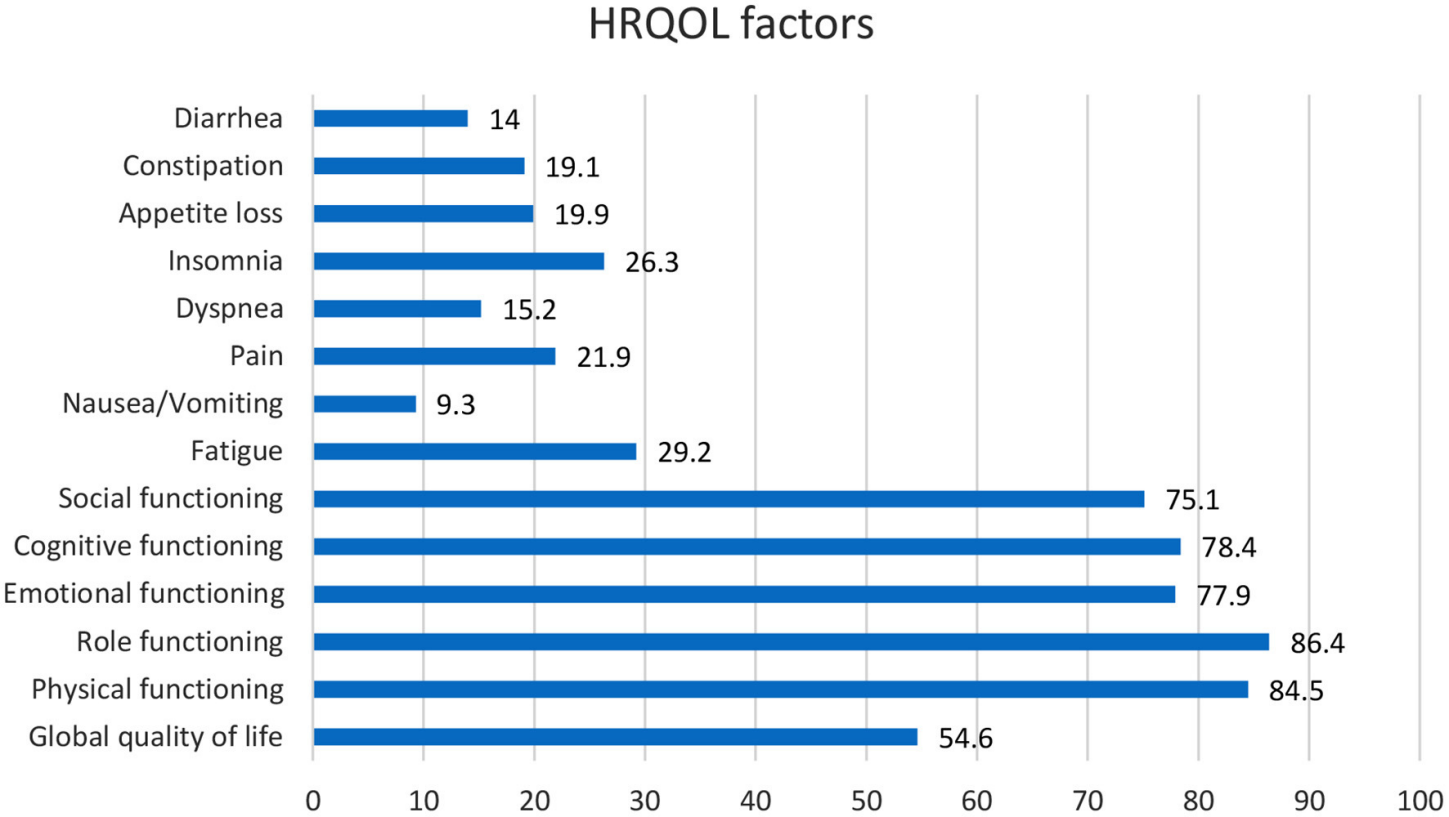
According to the information in an article entitled head and Neck Cancer Patients' Quality of Life, we understood that HR-QoL issues contain five functional scales (physical, role, cognitive, emotional, and social), three symptom scales (fatigue, pain, and nausea/vomiting) and six single-items (dyspnea, insomnia, appetite loss, constipation, diarrhea, and financial difficulties).

**Figure 2:**
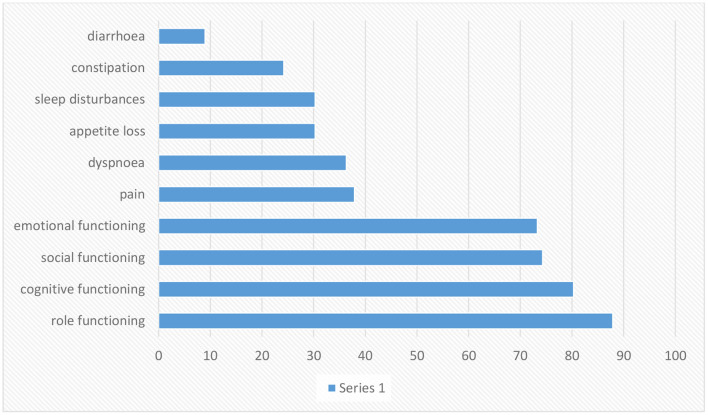
Scaling is based on article data entitled Experiences of daily Life and life quality in men with prostate cancer, which completely depicts the main findings in this regard (34).

The head and neck are two of the most significant vital diagnostic parts of the body of their complex structure and many physical procedures (24). Head and Neck cancer (HNC) references a group associated with tumors of the nasal cavity, oral cavity, throat, larynx, middle ear, and sinuses (25). HNC's risk factors include poor health in the oral cavity, environmental pollutants, gastroesophageal reflux illness, nutritional issues, and the usage of marijuana (26). One of the most important causes of HNC cancer is the use of chromium in different areas (27). The signs of this cancer might contain a swelling or pain that does not relent, a sore throat that does not resolve, trouble swallowing, and a variation or roughness in the voice (28). Health-associated matters are among the several issues that might affect QoL. Subsequently, HNC influences bodily structures that endanger everyday actions like talking, deglutition and breathing, drinking, and treatment might terminate abnormalities that harmfully affect psychosocial functioning (29). There was a confined decline of physical and role functioning and several head and neck symptoms in the first months of the disease, with development subsequently (Figure 1). But after a long illness duration, only physical functioning, taste/smell, dry mouth, and sticky saliva were significantly worse, contrasted with baseline. As women developed through the stages of cancer, and when combined with treatment it was related to additional symptoms, and worse functioning (30). The functional cognitive, physical, and emotional scales were the most affected. Pain, fatigue, and sleep disorders were the most widespread symptoms (31) (Figure 1). Head and neck cancers can change the appearance of patients because of their tumors, which in turn cause more emotional damage than other cancers (32). HNC patients experience weight changes owing to the disease (33) (Figure 2). According to an article entitled head and neck cancer patients' quality of life, health-related life quality factors have been included in Figure 1 below. The patients studied in the report are patients who have been identified through HNC and are experiencing antineoplastic treatment to treat cancer. The cure is comprehensive for 6 months. These patients were employed for expediency in the section of dentistry of the Mato Grosso Cancer Hospital, Cuiabá, MT, Brazil (35). Scaling is based on article data entitled head and Neck Cancer Patients' Quality of Life. Quality of life analysis of patients with head and neck cancer using the UW-QoL, EORTC QLQ-C30/QLQH&N 35, and FACT-H&N instruments expressed by mean, median, and standard deviation (35).

## Prostate Cancer

Prostate cancer morbidity and fatality measures often diverge. The second most common reason for cancer death in men is prostate cancer (36). There are no primary symptoms for most cases, and then late symptoms might include exhaustion due to anemia, bone ache, paralysis from spinal metastases, and renal failure from the bilateral ureteral obstacle (37). The danger of rising prostate cancer is related to advancing age, African American heritage, and positive family history of the disease, and maybe increased by diet and other influences (36). The risk factors that increase the advance of prostate cancer include family history, race, socioeconomic issues, occupation, infectious agents, sexual behavior, cadmium exposure, vasectomy, and smoking (38). Some kinds of prostate cancer progress slowly and might require very little or even no treatment; others are destructive and spread fast (39). Prostate cancer (PC) is the sixth most common kind of cancer among men universally, realized as a communal health issue worldwide (40–42). QoL is important for patients with prostate cancer; besides, the progression of the disease is relatively slow, which gives the hope of a long survival time (43). HRQoL covers the entire range of the human experience, including daily necessities, social relations, physical and psychological health, disease and job, and individual enjoyment (44, 45). Prostate cancer influences the bowels, bladder, and sexual role that affects the QoL (43). One of the fundamental problems in patients with prostate cancer is impotence. Other side effects of invasive cures include urinary and digestive dysfunction. Exhaustion, pain, hopelessness, marital disorders, nervousness, and worry around the progression of the illness affect the patient's quality of life (46). Men have to live with their disease and the side effects of treatment, which are primarily urinary, sexual, and gastral complications (47–49). Prostate cancer affects the older public, and deaths from this cancer are seen in the elderly and even middle-aged population (50). Patients with early stage prostate cancer need to comprehend the possible influence on their QoL from various treatments so they can choose the cure that fits their priorities and preferences (51). Incontinence and impotence increase after radiation therapy and radical prostatectomy, but radiation therapy rates are relatively lower (50). According to the figure, role functioning has been most affected and loss of appetite is also associated with diarrhea.

## Breast Cancer

**Figure 3:**
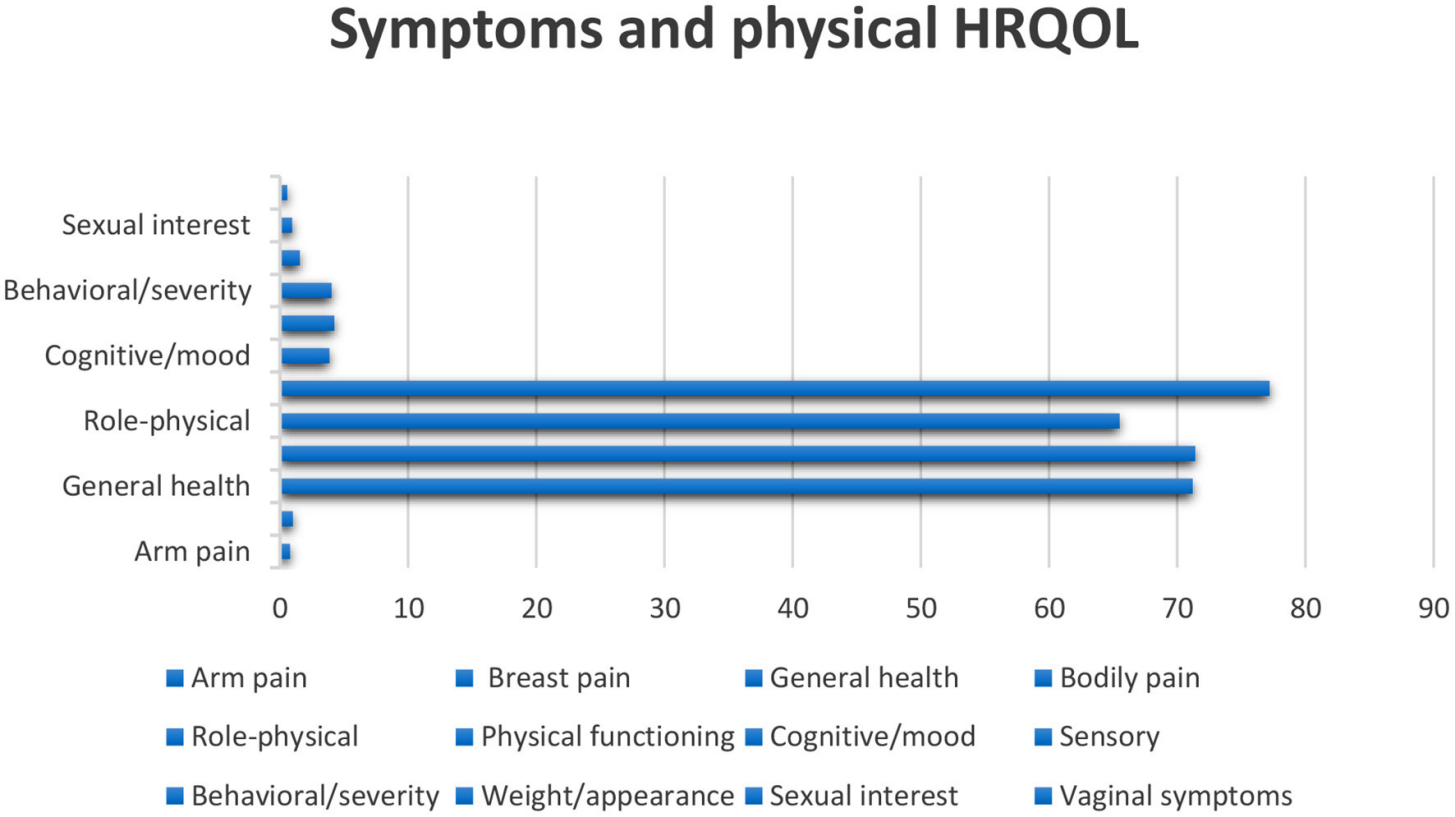
According to the above figure, we assessed some of the symptoms and some parts of HRQoL.

**Figure 4:**
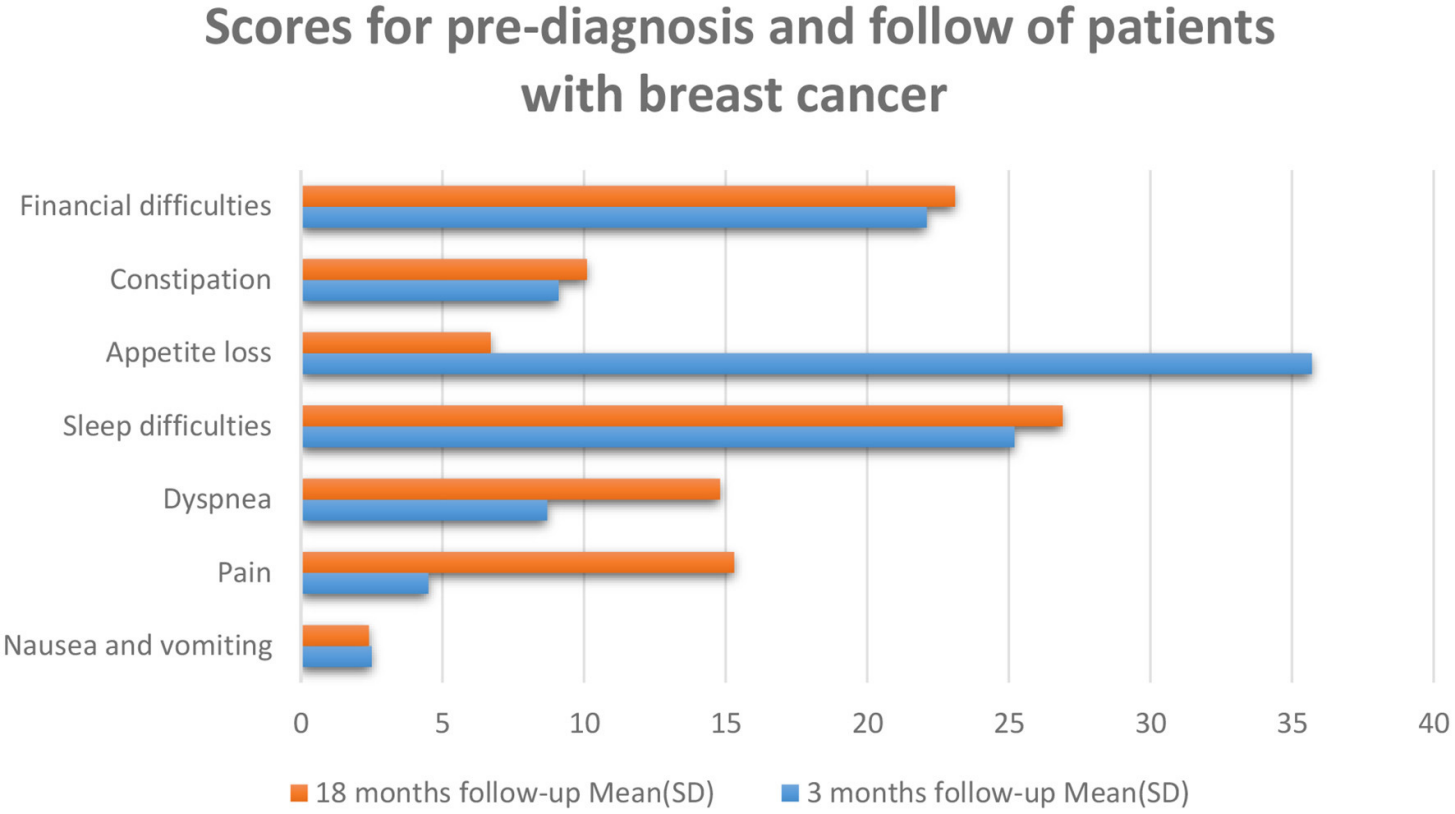
The patients at 18 months follow-up described reduced QoL. QoL 3 months are the resulting cure for breast cancer patients exposed to which there was reasonable suffering due to the anxiety of cancer reappearance and restarting everyday life. There were raised levels of fatigue, pain, and dyspnea at 18 months following a valuation. The higher values indicate a greater degree of symptoms, min: 0, max: 100 (66).

After skin cancer, breast cancer is the most common cancer among women and is the second greatest cause of cancer death in women after lung cancer (52). Some kinds of tumors might advance inside diverse parts of the breast and most tumors are the consequence of benign variations inside the breast. Breast cancer is much more common in women than men; and outcomes are not promising if there is a delay in diagnosis of the disease (53). In breast cancer patients, the mortality rate increases with age, and improved physical activity can decrease the danger of this cancer in women (54). Probable biological mechanisms that occur under the effect of physical activity on body composition contain insulin resistance and circulating stages of sex steroid hormones (54, 55). Breast cancer diagnosis methods involve a physical check, imaging—particularly mammography, and tissue biopsy. Early diagnosis and timely initiation of treatment will help cancer patients survive (56). HRLQoL was described through the way of breast cancer patients' understanding of their physical, emotional, and social well-being influenced by diagnosis, treatment, post-treatment, and survivorship as evaluated using well-validated tools (57) (Figure 3). Psychosocial issues complicate the signs of physical symptoms and affect the QoL of breast cancer patients (58) (Figure 3). Mental treatments can assist breast cancer patients in handling their feelings and mental issues they might cause, including depression, phobias, and anxieties (58, 59). Body deformity, sexual dysfunction, and syndromes that grow in advanced cancer patients after mastectomy affect QoL (57). The conclusion of treatment can remarkably trouble women with breast cancer, particularly those who have received adjuvant chemotherapy or radiation therapy. Signs such as hot flashes, oversleeping, and exhaustion reduce the QoL in women throughout breast cancer (60–65). Patients studied in an article by Ali Montazeri et al. were patients with a new diagnosis of breast cancer who have been admitted to Imam Khomeini Hospital in Tehran. Patients were evaluated in 2 stages; the introductory period was linked to 3 months after the early treatment, and the next period was made 1 year later (18 months subsequent to the pre-diagnosis) (66) (Figure 4). Eventually, the highlighted data from the previously mentioned research clearly and preciously show a linear relationship between the duration of follow-up of breast cancer patients, which has an indispensable role in evaluating QoL. Scaling this figure based on the data in the article entitled Physical activity, long-term symptoms, and physical health-related quality of life among breast cancer survivors: a prospective analysis, and according to the article signs besides physical HRQoL consequences stated by 545 breast cancer survivors (67).

## Lung Cancer

**Figure 5:**
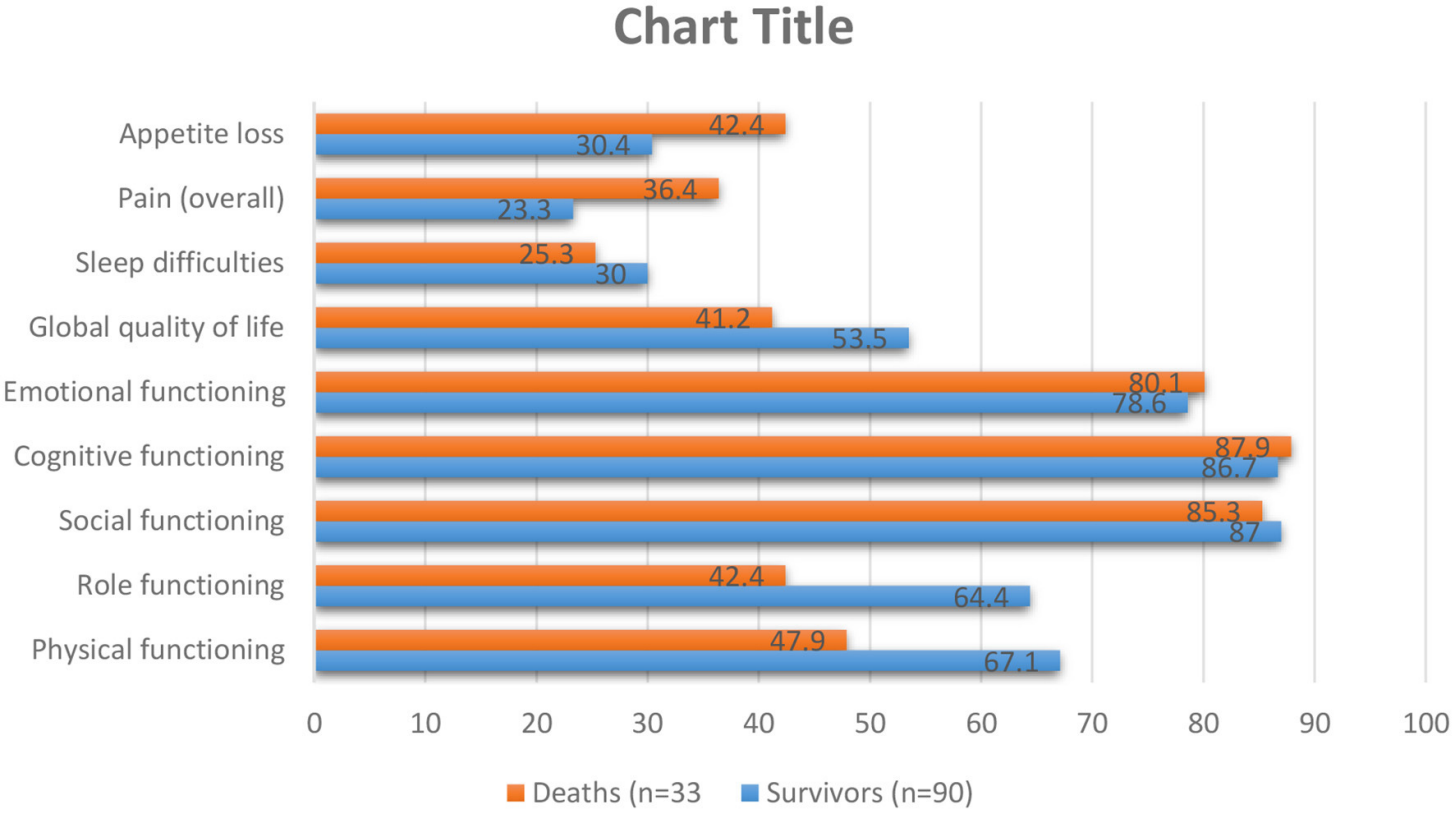
As shown in the table above, pre-diagnosis behavior besides global QoL were assessed for lung cancer patients for survivors then patients who pass away in the first months of diagnosis. The higher standards show a higher step of functioning and QoL: min., 0; max., 100. S.E.M.=usual error of the mean (82).

Lung cancer is the most common cancer incidence and cause of cancer mortality in men, while in women, it is the third most frequent cancer (68). Although lung cancer is separated into many subgroups, there are two primary sorts of lung cancer: non-small cell lung cancer (NSCLC) and small cell lung cancer. Smoking increases the risk of lung cancer, but non-smokers can also get it and by comparing these two types of lung cancer, we concluded that small cell lung cancer grows and spreads faster than non-small cell lung cancer (69). Lung cancer is often undiagnosed in the early stages because its symptoms may be similar to those of the common cold (69). Lung cancer can be very heterogeneous and can occur in different parts of the bronchial tree, so there are very variable signs and symptoms depending on its anatomical location. Lung cancers small cells are the most distinctive highly invasive cancers that spread rapidly to the lymph vessels beneath the mucosa and lymph nodes in the area (70). Risk factors for lung cancer include smoking, family history, exposure to secondhand smoke, mineral and metal particles, or asbestos. Symptoms of NSCLC can include cough, chest pain, shortness of breath, blood in sputum, wheezing, hoarseness, recurrent chest infections, weight loss, lack of appetite, and fatigue (71). QoL in patients with lung cancer is one of the most critical factors in prognosis quality of life by way of a predictive aspect. The main aspects of a patient's health may be negatively influenced through the diagnosis of cancer or its treatment (57). Symptoms of lung cancer significantly affect the patient's QoL, which significantly impacts physical, emotional, social, and mental health (72) (Figure 4). Increased exhaustion, shortness of breath, cough, and emotional distress decrease the quality of life, while difficulties with night-time rest affect cognitive function (73, 74). Worry and depression rise throughout chemotherapy, which affects the quality of life and the severity of symptoms (73). Patients' physical function is severely reduced after surgery, affecting patients' quality of Life (75–81). Research done by R Milroy et al. was based on two patient groups categorized into survivors and deaths. The survivors were those who lived for at least three-months, and the dead were those who lost their lives (82) (Figure 5). According to previously highlighted research, QoL assessment and comparison among survivors and fatalities can depict valuable information to realize better assessment factors that transparently show a bit different in their value allocation.

## Skin Cancer

**Figure 6:**
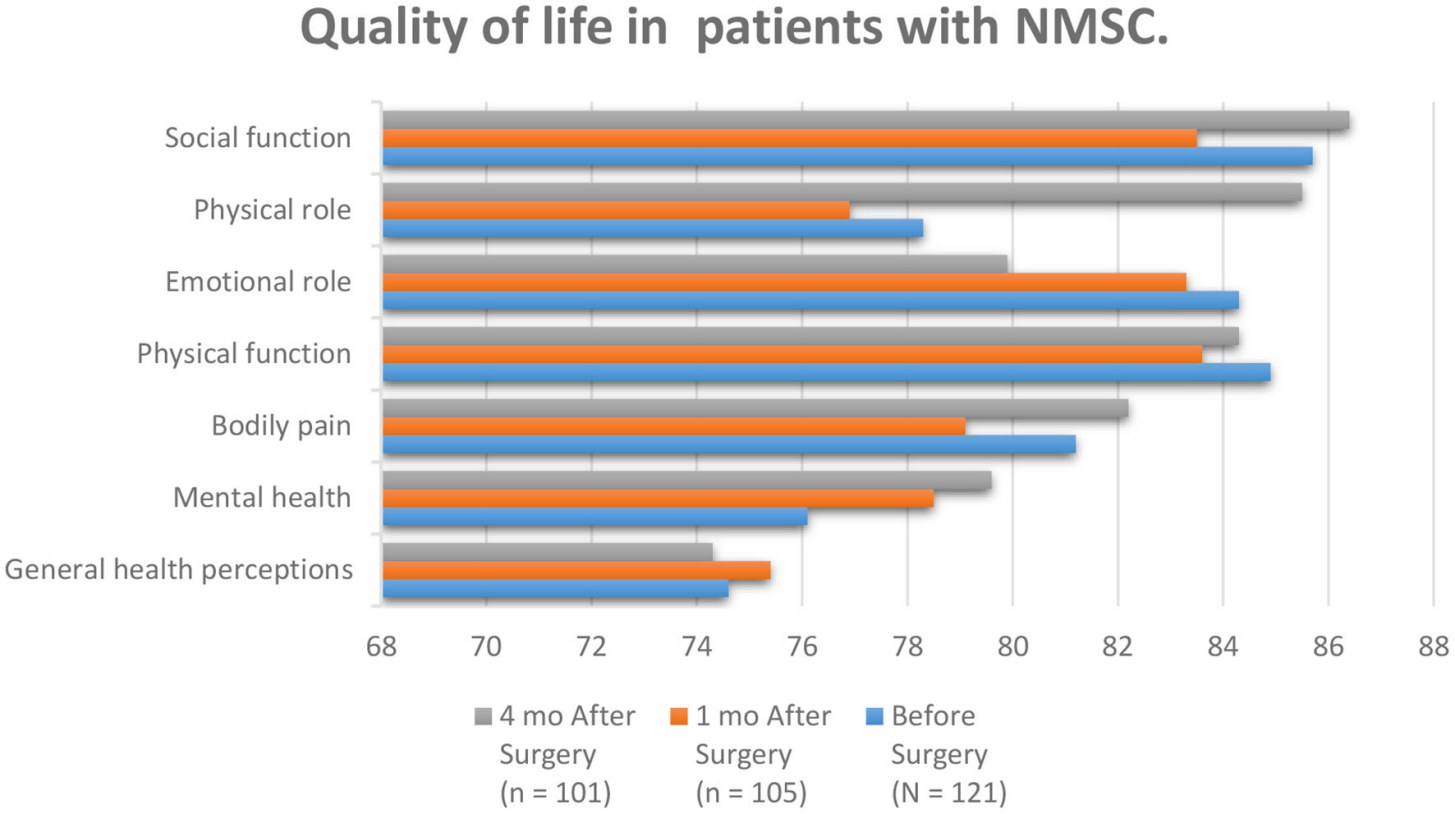
According to the patients studied in the article entitled quality of life and Sun-Protective behavior in Patients with skin cancer, the whole subscale generally scores were fairly high. Social functioning, physical role, and mental health improved compared to other scales after surgery (93).

Skin cancer is the most common cancer, and its occurrence is growing as young people expose themselves to vast amounts of ultraviolet radiance (UV) and use minimal skin defense, increasing their danger (83–90). Melanoma and no melanoma skin cancer (NMSC) are the most common kinds of cancer in white people at present. Equally, tumor entities display a growing occurrence amount universal than a constant or reducing humanity amount. NMSC is an ever-increasing problem for health services everywhere which causes a critical disease. The increasing occurrence charges of NMSC can be produced by a mixture of increased exposure to ultraviolet or sunlight, increased outdoor activity, variations in dress style, improved endurance, ozone reduction in the atmosphere, and genetics and in related cases, immunosuppression. Concentrated UV exposure in infants and youth caused the advance of basilar cell carcinoma (BCC) but for the etiology of SCC, lasting ultraviolet radiance exposure in the previous periods was suspect (91). Skin cancer is one of the most common malignancies, affecting patients' quality of life through a quickly accelerating occurrence each year. The QoL of the patients through skin cancer is influenced by the danger of complications, operation, and beauty and practical considerations. The influences that affect the QoL in patients with skin cancer are the diagnosis of the disease, surgical interposition, and scars. It must be emphasized that most NMSC appears on sun-exposed parts, such as the face, neck, and upper limbs (92). Patients through poor quality of life displayed further threatening cognitive and emotional disease signs, lower perceived communal care, advanced mental complications, and advanced anxiety related to body image (Figure 6). Body image mediated the relationship among mental and understanding disease representations, family pressure, mental difficulties, and quality of life (94). It is clear that among seven factors of QoL mentioned in Figure 6, four out of seven show improvement after surgery in comparison with before surgery which demonstrates condition advancement of NMSC patients.

## Discussion

We assess and compare the data in the figures of HNC and PC, according to the figures, head and neck cancer affects the role and cognitive function more than prostate cancer. Prostate cancer patients have less appetite than head and neck cancer patients, and affording to the figures, dyspnea is more common in prostate cancer patients than in head and neck cancer patients. The social and emotional function is more affected in prostate cancer, and pain intensity is felt more in head and neck cancer patients than in prostate cancer patients. In both diseases, patients develop diarrhea, but in patients with head and neck cancer, there is more than in patients with prostate cancer. Based on prostate and lung cancer figures, we compare the quality of life of prostate and lung cancer patients. The effect of prostate cancer on the social, cognitive, and emotional functioning of patients is also greater, the severity and amount of pain in prostate cancer patients are higher than in lung cancer patients. Sleep disturbance is seen in both diseases, but they are almost more elevated in prostate cancer patients. Also, in both disorders, the appetite is decreased. Comparing the quality of life of patients with these two cancers, we concluded that the severity of pain that skin cancer patients suffer is much higher than that of head and neck cancer patients. However, skin cancer patients who have not yet had surgery have better mental health, but comparing the data, we concluded that physical function is almost the same in both diseases, but the social part of head and neck cancer patients is better than skin cancer. In patients with prostate cancer, gastrointestinal disorders such as constipation and diarrhea are observed, and even the patient's sleep is disturbed. Prostate cancer has a more negligible effect on functional scales such as social and emotional than skin cancer. Comparing the quality of life of skin cancer with lung cancer, we found that the impact of lung cancer on the patient's physical, social, and emotional functioning is more significant than skin cancer. On the other hand, the severity of body pain in skin cancer patients is higher, although, by comparing breast cancer with other cancers, we found that the intensity of pain that breast cancer patients endure is lower than that of patients with head and neck and skin cancer. According to the data in the figures, nausea and vomiting are lower in breast cancer patients than in head and neck cancer patients. The severity of dyspnea in breast cancer patients is less than that of head and neck cancer. Sleep difficulties experienced by lung cancer survivors and prostate cancer patients are greater than those experienced by breast cancer patients. Comparing the figures, it was concluded that appetites in breast cancer patients in the first 3 months of the disease were reduced more than in patients with head and neck, prostate, and lung cancer. According to the information in the tables, we examined the dimensions of physical functioning and role functioning, social functioning, and emotional functioning among the cancers mentioned in this article, namely head and neck cancer and prostate cancer, breast cancer, lung, and skin cancer, and came to this conclusion. We found that the physical functioning dimension in head and neck cancer is more favorable than other cancers and the physical functioning of head and neck cancer patients is better than other cancers, and this dimension is less affected in head and neck cancer patients. Role functioning among prostate cancer patients is better than other cancer patients in this article. Also, among cancers, lung cancer has the most significant impact on physical functioning and role functioning. Examining the dimensions of emotional function and social function, it can be said that these two dimensions are more affected by head and neck cancer than other cancers, while the social role of lung cancer patients and the emotional function of skin cancer patients were better than the others.

We have added four more cancers to this article to expand the research results, and they are shown in the table below. According to the information in the tables, we examined the dimensions of physical functioning, emotional functioning, social functioning, cognitive functioning, role functioning, nausea and vomiting, appetite loss, fatigue, pain, dyspnea, and diarrhea among cancers mentioned in this article, namely head and neck cancer, prostate cancer, breast cancer, lung, skin cancer, cervical cancer, colon cancer, stomach cancer, and esophageal cancer, and concluded. Regarding the four additional cancers added to the table, the patients studied in colon cancer are patients who have undergone 3 months of rehabilitation program (95). But in the case of stomach cancer, the scales are evaluated by patients who have had a total gastrectomy (96). According to the patients studied in the article entitled, Quality of Life in Cervical Cancer Survivors and Healthy Women: Thai Urban Population Study, we were able to compare the scales in the table above with other scales. Quality of life survey through scales in the normal population in cancer of the esophagus is shown in Tables 1, 2 (98).

**Table 1:** This table shows which scales are considered for each cancer in this article.

**Cancers**	**Scales proposed for each cancer**
Head and neck cancer	Physical functioning, emotional functioning, social functioning, cognitive functioning role functioning, nausea & vomiting, appetite loss, fatigue, pain, dyspnea, diarrhea
Prostate cancer	Emotional functioning, social functioning, cognitive functioning role functioning, appetite loss, pain, dyspnea, diarrhea
Breast cancer	Cognitive functioning, role functioning, nausea & vomiting, appetite loss, pain, dyspnea
Lung cancer	Physical functioning, emotional functioning, social functioning, cognitive functioning role functioning, appetite loss, pain
Skin cancer	Physical functioning, emotional functioning, social functioning, role functioning, pain
Cervical cancer	Physical functioning, emotional functioning, social functioning, cognitive functioning role functioning, nausea & vomiting, appetite loss, fatigue, pain, dyspnea, diarrhea (97)
Colon cancer	Physical functioning, emotional functioning, social functioning, cognitive functioning role functioning, nausea & vomiting, appetite loss, fatigue, pain, dyspnea, diarrhea (95)
Stomach cancer	Physical functioning, emotional functioning, social functioning, cognitive functioning role functioning, nausea & vomiting, appetite loss, fatigue, pain, dyspnea, diarrhea (96)
Esophageal cancer	Physical functioning, emotional functioning, social functioning, role functioning, fatigue, pain (98)

**Table 2:** A comparison of all the parameters in all the cancers mentioned in this article is prepared in the table above, which shows for each criterion which cancer is highest and which is lowest.

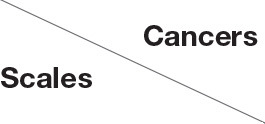	**Head and neck cancer**	**Prostate cancer**	**Breast cancer**	**Lung cancer**	**Skin cancer**	**Cervical cancer**	**Colon cancer**	**Stomach cancer**	**Esophageal cancer**
Physical functioning									
Emotional functioning									
Social functioning									
Cognitive functioning									
Role functioning									
Nausea and vomiting									
Appetite loss									
Fatigue									
Pain									
Dyspnea									
Diarrhea									

*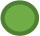 Maximum*.

*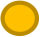 Minimum*.

## Conclusion

Reviewing and researching the Quality of Life of patients can give us essential information about patients' responses to cancer and cancertreatments, and in the communication of diverse responses and the general Quality of Life, the data gained can also affect the care options chosen. As a result, the most critical cancers encompassing breast, head, neck, prostate, lung, and skin cancer were the topical parameters and important items discussed in this article. It can be found that side effects of treatment procedures affect quality-of-life parameters efficiently. Symptoms of cancer can significantly impact the quality of life, which substantially impacts physical, emotional, social, and spiritual health. In general, emotional and physical anxiety and fear of cancer, changes in the patient's appearance, and loss of the patient's ability to perform daily tasks can significantly impact patients' Quality of Life.

## Author Contributions

SJ proposed the main idea and gathered data and RG revised the article. Both authors contributed to the article and approved the submitted version.

## Conflict of Interest

The authors declare that the research was conducted in the absence of any commercial or financial relationships that could be construed as a potential conflict of interest.

## Publisher's Note

All claims expressed in this article are solely those of the authors and do not necessarily represent those of their affiliated organizations, or those of the publisher, the editors and the reviewers. Any product that may be evaluated in this article, or claim that may be made by its manufacturer, is not guaranteed or endorsed by the publisher.

## Abbreviations

QoL: Quality of life
HNC: Head and neck cancer
HRQoL: Health-related quality of life
PC: Prostate cancer
NSCLC: non-small cell lung cancer
SCLS: Small cell lung cancer
NMSC: No melanoma skin cancer
BCC: Basilar cell carcinoma
PC: Prostate cancer.

## References

[1] Liu H Zeng H Shen Y Zhang F Sharma M Lai W. Assessment tools for health literacy among the general population: a systematic review. Int J Environ Res Public Health. (2018) 15:1711. 10.3390/ijerph15081711 30103386 PMC6122038

[2] Fernald DH Tsai AG Vance B James KA Barnard J Staton EW. Health Assessments in Primary Care: A How-to-Guide for Clinicians Staff. Health Assess Primary Care. No. 13-0061-EF. (2020). Available online at: https://www.ahrq.gov/sites/default/files/publications/files/health-assessments_0.pdf

[3] Stevens A Gillam S. Needs assessment: from theory to practice. BMJ. (1998) 316:1448–52. 10.1136/bmj.316.7142.1448 9572762 PMC1113121

[4] de Haes JCJM van Knippenberg FCE. The quality of life of cancer patients: a review of the literature. Soc Sci Med. (1985) 20:809–17. 10.1016/0277-9536(85)90335-1 3890194

[5] Allen PF. Assessment of oral health related quality of life. Health Q Life Outcomes. (2003) 1:40. 10.1186/1477-7525-1-40 14514355 PMC201012

[6] Guyatt GH Feeny DH Patrick DL. Measuring health-related quality of life. Ann Internal Med. (1993) 118:622–9. 10.7326/0003-4819-118-8-199304150-00009 8452328

[7] Donovan K Sanson-Fisher RW Redman S. Measuring quality of life in cancer patients. J Clin Oncol. (1989) 7:959–68. 10.1200/JCO.1989.7.7.959 2661736

[8] Stehlin Jr JS Beach KH. Psychological aspects of cancer therapy: a surgeon's viewpoint. JAMA. (1966) 197:100–4. 10.1001/jama.197.2.100 5952488

[9] Silberfarb PM. Psychiatric problems in breast cancer. Cancer. (1984) 53:820–4. 10.1002/1097-0142(19840201)53:3+<820::aid-cncr2820531335>3.0.co;2-4 6692281

[10] Calman KC. Quality of life in cancer patients–an hypothesis. Journal of Medical Ethics. (1984) 10:124. 10.1136/jme.10.3.124 6334159 PMC1374977

[11] Brown LF Kroenke K Theobald DE Wu J Tu W. The association of depression and anxiety with health-related quality of life in cancer patients with depression and/or pain. Psychooncology. (2010) 19:734–41. 10.1002/pon.1627 19777535 PMC2888919

[12] Iconomou G Mega V Koutras A Iconomou AV Kalofonos HP. Prospective assessment of emotional distress, cognitive function, and quality of life in patients with cancer treated with chemotherapy. Cancer. (2004) 101:404–11. 10.1002/cncr.20385 15241840

[13] Ahles TA Saykin AJ Furstenberg CT Cole B Mott LA Skalla K. Neuropsychologic impact of standard-dose systemic chemotherapy in long-term survivors of breast cancer and lymphoma. J Clin Oncol. (2002) 20:485–93. 10.1200/JCO.2002.20.2.485 11786578

[14] Ahles TA Saykin A. Cognitive effects of standard-dose chemotherapy in patients with cancer. Cancer Invest. (2001) 19:812–20. 10.1081/CNV-100107743 11768035

[15] McDaniel JS Musselman DL Porter MR Reed DA Nemeroff CB. Depression in patients with cancer. Diagnosis, biology, and treatment. Arch Gen Psychiatry. (1995) 52:89–99. 10.1001/archpsyc.1995.03950140007002 7848055

[16] Baker F Haffer SC Denniston M. Health-related quality of life of cancer and noncancer patients in medicare managed care. Cancer. (2003) 97:674–81. 10.1002/cncr.11085 12548610

[17] Byers T Mouchawar J Marks J Cady B Lins N Swanson GM. The American cancer society challenge goals. How far can cancer rates decline in the U.S. by the year 2015? Cancer. (1999) 86:715–27. 10.1002/(SICI)1097-0142(19990815)86:4&lt;715::AID-CNCR22&gt;3.0.CO;2-O 10440701

[18] Osoba D. Health-related quality of life and cancer clinical trials. Therapeutic Adv Med Oncol. (2011) 3:57–71. 10.1177/1758834010395342 21789156 PMC3126042

[19] Nayak MG George A Vidyasagar MS Mathew S Nayak S Nayak BS. Quality of life among cancer patients. Indian J Palliative Care. (2017) 23:445–50. 10.4103/IJPC.IJPC_82_17 29123353 PMC5661349

[20] Maughan TS James RD Kerr DJ Ledermann JA McArdle C Seymour MT. British MRC Colorectal Cancer Working Party, Comparison of survival, palliation, and quality of life with three chemotherapy regimens in metastatic colorectal cancer: a multicentre randomised trial. Lancet. (2002) 359:1555–63. 10.1016/S0140-6736(02)08514-8 12047964

[21] de Jong N Candel MJJM Schouten HC Huijer Abu-Saad H Courtens AM. Prevalence and course of fatigue in breast cancer patients receiving adjuvant chemotherapy. Ann Oncol. (2004) 15:896–905. 10.1093/annonc/mdh229 15151946

[22] Lin XJ Lin IM Fan SY. Methodological issues in measuring health-related quality of life. Tzu Chi Med J. (2013) 25:8–12. 10.1016/j.tcmj.2012.09.00219860820

[23] Khalilnejad M Mortezazadeh T Ghasemi Shayan R. Application of Manganese Oxide (MnO) nanoparticles in multimodal molecular imaging and cancer therapy: a review. Nanomed J. (2021) 8:166–78. 10.22038/nmj.2021.57687.1598

[24] Ghasemi Shayan R Oladghaffari M Sajjadian F Ghaziyani MF. Image Quality and dose comparison of single-energy CT (SECT) and dual-energy CT (DECT). Radiol Res Practice. (2020) 2020:1403957. 10.1155/2020/1403957 32373363 PMC7189324

[25] Dunne S Mooney O Coffey L Sharp L Desmond D Timon C. Psychological variables associated with quality of life following primary treatment for head and neck cancer: a systematic review of the literature from 2004 to 2015. Psychooncology. (2017) 26:149–60. 10.1002/pon.4109 26918648

[26] Mao L Hong WK Papadimitrakopoulou VA. Focus on head and neck cancer. Cancer Cell. (2004) 5:311–6. 10.1016/S1535-6108(04)00090-X 15093538

[27] Sabonian M Mahanpoor K. Optimization of photocatalytic reduction of Cr(VI) in water with Nano ZnO/todorokite as a catalyst: using taguchi experimental design. Iranian J Chem Chem Eng. (2019) 38:105–13. 10.30492/ijcce.2019.32872

[28] Institute NC. Head Neck Cancers. National Cancer Institute. USA.gov. (2017).

[29] Murphy BA Ridner S Wells N Dietrich M. Quality of life research in head and neck cancer: a review of the current state of the science. Crit Rev Oncol /Hematol. (2007) 62:251–67. 10.1016/j.critrevonc.2006.07.005 17408963

[30] de Graeff A de Leeuw JR Ros WJ Hordijk GJ Blijham GH Winnubst JA. Long-term quality of life of patients with head and neck cancer. (2000). Laryngoscope. 110:98–106. 10.1097/00005537-200001000-00018 10646723

[31] Rigoni L Bruhn RF De Cicco R Kanda JL Matos LL. Quality of life impairment in patients with head and neck cancer and their caregivers: a comparative study. Braz J Otorhinolaryngol. (2016) 82:680–6. 10.1016/j.bjorl.2015.12.012 27133907 PMC9444724

[32] Björklund M Sarvimäki A Berg A. Living with head and neck cancer: a profile of captivity. J Nurs Healthcare Chronic Illness. (2010) 2:22–31. 10.1111/j.1752-9824.2010.01042.x

[33] Hassan SJ Weymuller Jr EA. Assessment of quality of life in head and neck cancer patients. Head Neck. (1993) 15:485–96. 10.1002/hed.2880150603 8253555

[34] Jakobsson L Hallberg IR Lovén L. Experiences of daily life and life quality in men with prostate cancer. An explorative study. Part I. Eur J Cancer Care (Engl). (1997) 6:108–16. 10.1046/j.1365-2354.1997.00019.x 9233161

[35] Gomes EPAdA Aranha AMF Borges AH Volpato LER. Head and neck cancer patients' quality of life: analysis of three instruments. J Dentistry. (2020) 21:31–41. 10.30476/DENTJODS.2019.77677.0 32158782 PMC7036356

[36] Haas GP Sakr WA. Epidemiology of prostate cancer. Cancer J Clin. (1997) 47:273–87. 10.3322/canjclin.47.5.273 9314822

[37] Leslie SW Soon-Sutton TL Sajjad H Sereif LE. Prostate Cancer. In: StatPearls [Internet]. Treasure Island, FL: StatPearls Publishing (2022). Available online at: https://www.ncbi.nlm.nih.gov/books/NBK470550/

[38] Pienta KJ Esper PS. Risk factors for prostate cancer. Ann Intern Med. (1993) 118:793–803. 10.7326/0003-4819-118-10-199305150-00007 8470854

[39] Staff MC. Prostate Cancer. Mayo Clinic (2020).

[40] Maringá JPEv. Prostate cancer: quality of life and physical activity level of patients. J Phys Educ. (2018) 29:e2932. 10.4025/jphyseduc.v29i1.2932

[41] Silva AA de Andrade CAC Antunes CE de Souza PF da Silva NRM. Strategies for the prevention of prostate cancer. J Revista de Pesquisa. (2013). 10.9789/2175-5361.2013.v5i2.3795-3807

[42] Moscheta MdS Mad S. Grupos de apoio para homens com câncer de próstata: revisão integrativa da literatura. J Ciência Saúde Coletiva. (2012). 17:1225–33. 10.1590/S1413-81232012000500016 22634815

[43] McPherson CP Swenson KK Kjellberg J. Quality of life in patients with prostate cancer. Semin Oncol Nurs. (2001) 17:138–46. 10.1016/S0749-2081(01)80021-8 11383245

[44] Bergman J Laviana A. Quality-of-life assessment tools for men with prostate cancer. Nat Rev Urol. (2014) 11:352–9. 10.1038/nrurol.2014.101 24841165

[45] Patrick DL Erickson P. Assessing health-related quality of life for clinical decision-making. In: Walker SR Rosser RM editors. Quality of Life Assessment: Key Issues in the 1990s. Dordrecht: Springer Netherlands (1993). p. 11–63. 10.1007/978-94-011-2988-6_2

[46] Giesler RB Given B Given CW Rawl S Monahan P Burns D. Improving the quality of life of patients with prostate carcinoma. Cancer. (2005) 104:752–62. 10.1002/cncr.21231 15986401

[47] Schmidt S Garin O Pardo Y Valderas JM Alonso J Rebollo P. Assessing quality of life in patients with prostate cancer: a systematic and standardized comparison of available instruments. Q. Life Res. (2014) 23:2169–81. 10.1007/s11136-014-0678-8 24748557 PMC4155169

[48] Sanda MG Dunn RL Michalski J Sandler HM Northouse L Hembroff L. Quality of life and satisfaction with outcome among prostate-cancer survivors. N Engl J Med. (2008) 358:1250–61. 10.1056/NEJMoa074311 18354103

[49] Miller DC Sanda MG Dunn RL Montie JE Pimentel H Sandler HM. Long-term outcomes among localized prostate cancer survivors: health-related quality-of-life changes after radical prostatectomy, external radiation, and brachytherapy. J Clin Oncol. (2005) 23:2772–80. 10.1200/JCO.2005.07.116 15837992

[50] Herr HW. Quality of life in prostate cancer patients. CA Cancer J Clin. (1997) 47:207–17. 10.3322/canjclin.47.4.207 9242169

[51] Talcott JA. Quality of life in prostate cancer. Eur J Cancer. (2005) 30:302–8. 10.1016/j.ejca.2004.12.030 15808958

[52] Ely S Vioral AN. Breast cancer overview. Plast Surg Nurs. (2007) 27:128–33; quiz 134–5. 10.1097/01.PSN.0000290281.48197.ae 17901821

[53] Sharma GN Dave R Sanadya J Sharma P Sharma KK. Various types and management of breast cancer: an overview. J Adv Pharm Technol Res. (2010) 1:109–26. 22247839 PMC3255438

[54] Coughlin SS. Epidemiology of breast cancer in women. Adv Exp Med Biol. (2019) 1152:9–29. 10.1007/978-3-030-20301-6_2 31456177

[55] Farvid MS Chen WY Michels KB Cho E Willett WC Heather Eliassen A. Fruit and vegetable consumption in adolescence and early adulthood and risk of breast cancer: population based cohort study. BMJ. (2016) 353:i2343. 10.1136/bmj.i2343 27170029 PMC5068921

[56] Alkabban FM Ferguson T. Breast cancer. In: StatPearls. StatPearls Publishing Copyright © (2021). Treasure Island, FL: StatPearls Publishing LLC (2021).

[57] Mokhatri-Hesari P Montazeri A. Health-related quality of life in breast cancer patients: review of reviews from 2008 to 2018. Health Q Life Outcomes. (2020) 18:338. 10.1186/s12955-020-01591-x 33046106 PMC7552560

[58] Perry S Kowalski TL Chang CH. Quality of life assessment in women with breast cancer: benefits, acceptability and utilization. Health Q Life outcomes. (2007) 5:24–24. 10.1186/1477-7525-5-24 17474993 PMC1877797

[59] Institute of Medicine (US) and National Research Council (US) National Cancer Policy Board. Meeting Psychosocial Needs of Women with Breast Cancer. In: Hewitt M Herdman R Holland J editors. Washington, DC: National Academies Press (2004). 25009861

[60] Paraskevi T. Quality of life outcomes in patients with breast cancer. Oncol Rev. (2012) 6:e2–e2. 10.4081/oncol.2012.22 25992204 PMC4419638

[61] Ward SE Viergutz G Tormey D deMuth J Paulen A. Patients' reactions to completion of adjuvant breast cancer therapy. Nurs Res. (1992) 41:362–6. 10.1097/00006199-199211000-00008 1437586

[62] Fertig DL. Depression in patients with breast cancer: prevalence, diagnosis, and treatment. Breast J. (1997) 3:292–302. 10.1111/j.1524-4741.1997.tb00184.x

[63] Holland JC Rowland JH. (Eds.). Handbook of Psychooncology: Psychological Care of the Patient With Cancer. Oxford: Oxford University Press (1989).

[64] Beisecker A Cook MR Ashworth J Hayes J Brecheisen M Helmig L. Side effects of adjuvant chemotherapy: perceptions of node-negative breast cancer patients. Psychooncology. (1997) 6:85–93. 10.1002/(SICI)1099-1611(199706)6:2<85::AID-PON247>3.0.CO;2-T 9205966

[65] DeSantis C Siegel R Bandi P Jemal A. Breast cancer statistics, 2011. CA Cancer J Clin. (2011) 61:409–18. 10.3322/caac.20134 21969133

[66] Montazeri A Vahdaninia M Harirchi I Ebrahimi M Khaleghi F Jarvandi S. Quality of life in patients with breast cancer before and after diagnosis: an eighteen months follow-up study. BMC cancer. (2008) 8:330–0. 10.1186/1471-2407-8-330 19014435 PMC2588619

[67] Alfano CM Smith AW Irwin ML Bowen DJ Sorensen B Reeve BB. Physical activity, long-term symptoms, and physical health-related quality of life among breast cancer survivors: a prospective analysis. J Cancer Surviv. (2007) 1:116–28. 10.1007/s11764-007-0014-1 18648952 PMC2996230

[68] William D Travis M. Pathology of lung cancer. Clin Chest Med. (2011) 32:669–92. 10.1016/j.ccm.2011.08.005 22054879

[69] Pietrangelo A. Everything you need to know about lung cancer. Healthline. (2021).

[70] Lemjabbar-Alaoui H Hassan OU Yang YW Buchanan P. Lung cancer: biology and treatment options. Bioch Biophys Acta. (2015) 1856:189–210. 10.1016/j.bbcan.2015.08.002 26297204 PMC4663145

[71] Sabbula BR Anjum F. Squamous Cell Lung Cancer. In: StatPearls [Internet]. Treasure Island, FL: StatPearls Publishing (2022). Available online at: https://www.ncbi.nlm.nih.gov/books/NBK564510/

[72] Akin S Can G Aydiner A Ozdilli K Durna Z. Quality of life, symptom experience and distress of lung cancer patients undergoing chemotherapy. Eur J Oncol Nurs. (2010) 14:400–9. 10.1016/j.ejon.2010.01.003 20149733

[73] Polanski J Jankowska-Polanska B Rosinczuk J Chabowski M Szymanska-Chabowska A. Quality of life of patients with lung cancer. Oncotargets Therapy. (2016) 9:1023–8. 10.2147/OTT.S100685 27013895 PMC4778772

[74] Brown DJ McMillan DC Milroy R. The correlation between fatigue, physical function, the systemic inflammatory response, and psychological distress in patients with advanced lung cancer. Cancer. (2005) 103:377–82. 10.1002/cncr.20777 15558809

[75] Sarna L Padilla G Holmes C Tashkin D Brecht ML Evangelista L. Quality of life of long-term survivors of non–small-cell lung cancer. J Clin Oncol. (2002) 20:2920–9. 10.1200/JCO.2002.09.045 12089220

[76] Dales RE Bélanger R Shamji FM Leech J Crépeau A Sachs HJ. Quality-of-life following thoracotomy for lung cancer. J Clin Epidemiol. (1994) 47:1443–9. 10.1016/0895-4356(94)90088-4 7730853

[77] Nugent AM Steele IC Carragher AM McManus K McGuigan JA Gibbons JR. Effect of thoracotomy and lung resection on exercise capacity in patients with lung cancer. Thorax. (1999) 54:334–8. 10.1136/thx.54.4.334 10092695 PMC1745463

[78] Zieren HU Müller JM Hamberger U Pichlmaier H. Quality of life after surgical therapy of bronchogenic carcinoma. Eur J Cardio Thoracic Surg. (1996) 10:233–7. 10.1016/S1010-7940(96)80144-8 8740057

[79] Pelletier C Lapointe L LeBlanc P. Effects of lung resection on pulmonary function and exercise capacity. BMJ. (1990) 45:49−502. 10.1136/thx.45.7.497 2396230 PMC462576

[80] Epstein SK Faling LJ Daly BD Celli BR. Inability to perform bicycle ergometry predicts increased morbidity and mortality after lung resection. Chest. (1995) 107:311–6. 10.1378/chest.107.2.311 7842753

[81] Mangione CM Goldman L Orav EJ Marcantonio ER Pedan A Ludwig LE. Health-related quality of life after elective surgery. J Gen Internal Med. (1997) 12:686–97. 10.1046/j.1525-1497.1997.07142.x 9383137 PMC1497188

[82] Montazeri A Milroy R Hole D McEwen J Gillis CR. Quality of life in lung cancer patients: as an important prognostic factor. Lung Cancer. (2001) 31:233–40. 10.1016/S0169-5002(00)00179-3 11165402

[83] Carolyn J Heckman P. Efficacy of an intervention to alter skin cancer risk behaviors in young adults. Am J Prev Med. (2016) 51: 1–11. 10.1016/j.amepre.2015.11.008 26810358 PMC4914462

[84] Donaldson MR Coldiron BM. No end in sight: the skin cancer epidemic continues. Semin Cutan Med Surg. (2011) 30:3–5. 10.1016/j.sder.2011.01.002 21540015

[85] Gordon R. Skin cancer: an overview of epidemiology and risk factors. Semin Oncol Nurs. (2013) 29:160–9. 10.1016/j.soncn.2013.06.002 23958214

[86] Nikolaou V Stratigos AJ. Emerging trends in the epidemiology of melanoma. Br J Dermatol. (2014) 170:11–9. 10.1111/bjd.12492 23815297

[87] Health USDO Human S. Reports of the surgeon general. In: The Surgeon General's Call to Action to Prevent Skin Cancer. Washington, DC: Office of the Surgeon General (US) (2014). 25320835

[88] Tuong W Cheng LS Armstrong AW. Melanoma: epidemiology, diagnosis, treatment, and outcomes. Dermatol Clin. (2012) 30:1131–24, ix. 10.1016/j.det.2011.08.006 22117873

[89] Stanton WR Janda M Baade PD Anderson P. Primary prevention of skin cancer: a review of sun protection in Australia and internationally. Health Promot Int. (2004) 19:369–78. 10.1093/heapro/dah310 15306621

[90] MacNeal RJ Dinulos JG. Update on sun protection and tanning in children. Curr Opin Pediatr. (2007) 19:425–9. 10.1097/MOP.0b013e3282294936 17630607

[91] Razi S Enayatrad M Mohammadian-Hafshejani A Salehiniya H Fathali-Loy-Dizaji M Soltani S. The epidemiology of skin cancer and its trend in Iran. Int J Prev Med. (2015) 6:64. 10.4103/2008-7802.161074 26288708 PMC4521305

[92] Răducu L Avino A Purtan RP Balcangiu-Stroescu AE Bălan DG Timofte D. Quality of life in patients with surgically removed skin tumors. Medicina. (2020) 56:66. 10.3390/medicina56020066 32050413 PMC7074335

[93] Rhee JS Alex Matthews B Neuburg M. Quality of life and sun-protective behavior in patients with skin cancer. Arch Otolaryngol Head Neck Surg. (2004) 130:141–6. 10.1001/archotol.130.2.141 14967741

[94] Pereira MG Ponte M Ferreira G Machado JC. Quality of life in patients with skin tumors: the mediator role of body image and social support. Psychooncology. (2017) 26:815–821. 10.1002/pon.4236 27502437

[95] Lamprecht J Thyrolf A Mau W. Health-related quality of life in rehabilitants with different cancer entities. Eur J Cancer Care. (2017) 26:e12554. 10.1111/ecc.12554 27482937

[96] Bae JM Kim S Kim YW Ryu KW Lee JH Noh JH. Health-related quality of life among disease-free stomach cancer survivors in Korea. Qual Life Res. (2006) 15:1587–96. 10.1007/s11136-006-9000-8 17036253

[97] Prasongvej P Nanthakomon T Jaisin K Chanthasenanont A Lertvutivivat S Tanprasertkul C. Quality of life in cervical cancer survivors and healthy women: thai urban population study. Asian Pac J Cancer Prev. (2017) 18:385–9. 10.22034/APJCP.2017.18.2.385 28345784 PMC5454732

[98] McLarty AJ Deschamps C Trastek VF Allen MS Pairolero PC Harmsen WS. Esophageal resection for cancer of the esophagus: long-term function and quality of life. Ann Thor Surg. (1997) 63:1568–71. 10.1016/S0003-4975(97)00125-2 9205149

